# A Wheat Cinnamyl Alcohol Dehydrogenase TaCAD12 Contributes to Host Resistance to the Sharp Eyespot Disease

**DOI:** 10.3389/fpls.2016.01723

**Published:** 2016-11-16

**Authors:** Wei Rong, Meiying Luo, Tianlei Shan, Xuening Wei, Lipu Du, Huijun Xu, Zengyan Zhang

**Affiliations:** The National Key Facility for Crop Gene Resources and Genetic Improvement, Institute of Crop Science, Chinese Academy of Agricultural SciencesBeijing, China

**Keywords:** hexaploid wheat, cinnamyl alcohol dehydrogenase (CAD), *Rhizoctonia cerealis*, sharp eyespot-resistance, defense genes, monolignol biosynthesis-related genes

## Abstract

Sharp eyespot, caused mainly by the necrotrophic fungus *Rhizoctonia cerealis*, is a destructive disease in hexaploid wheat (*Triticum aestivum* L.). In *Arabidopsis*, certain cinnamyl alcohol dehydrogenases (CADs) have been implicated in monolignol biosynthesis and in defense response to bacterial pathogen infection. However, little is known about CADs in wheat defense responses to necrotrophic or soil-borne pathogens. In this study, we isolate a wheat CAD gene *TaCAD12* in response to *R. cerealis* infection through microarray-based comparative transcriptomics, and study the enzyme activity and defense role of TaCAD12 in wheat. The transcriptional levels of *TaCAD12* in sharp eyespot-resistant wheat lines were significantly higher compared with those in susceptible wheat lines. The sequence and phylogenetic analyses revealed that TaCAD12 belongs to IV group in CAD family. The biochemical assay proved that TaCAD12 protein is an authentic CAD enzyme and possesses catalytic efficiencies toward both coniferyl aldehyde and sinapyl aldehyde. Knock-down of *TaCAD12* transcript significantly repressed resistance of the gene-silenced wheat plants to sharp eyespot caused by *R. cerealis*, whereas *TaCAD12* overexpression markedly enhanced resistance of the transgenic wheat lines to sharp eyespot. Furthermore, certain defense genes (*Defensin, PR10, PR17c*, and *Chitinase1*) and monolignol biosynthesis-related genes (*TaCAD1, TaCCR*, and *TaCOMT1*) were up-regulated in the *TaCAD12*-overexpressing wheat plants but down-regulated in *TaCAD12*-silencing plants. These results suggest that TaCAD12 positively contributes to resistance against sharp eyespot through regulation of the expression of certain defense genes and monolignol biosynthesis-related genes in wheat.

## Introduction

Hexaploid wheat (*Triticum aestivum* L., AABBDD, common wheat) is one of the most widely cultivated and consumed food crops. The global demand for wheat and other foods will increase along with the constantly increasing world population. Environmental stresses and diseases often negatively affect wheat production. For example, sharp eyespot is a devastating soil-borne disease impacting wheat production globally (Chen et al., 2008, 2013; Hamada et al., 2011; Lemańczyk and Kwaśna, 2013). China is the largest epidemic region in the world, as exemplified by more than 8.1 million hectares of wheat affected by sharp eyespot since 2005 (Chen et al., 2013; Zhu et al., 2015). The necrotrophic fungus *Rhizoctonia cerealis* van der Hoeven is a major causal pathogen of sharp eyespot (Chen et al., 2013). Sharp eyespot manifests as “eye”-shaped lesions on basal stems and basal sheaths of infected wheat plants. The disease can destroy the transport tissues in stems of plants and block transportation of substances required for nutrition, leading to yield losses of ∼10–40%. Breeding resistant wheat varieties is an effective and environmentally safe approach to control diseases. However, the sharp eyespot resistance in wheat accessions is partial and quantitative (Cai et al., 2006; Chen et al., 2013). To improve wheat resistance to sharp eyespot, it is vital to identify genes that play important roles in the defense response and unravel their underlying functional mechanisms. However, the complex and huge genome as well as transformation difficulty of common wheat make genetic and functional analyses extremely challenging.

To combat against invading microbial pathogens, plants have evolved a multi-layered immunity system. After plant recognition events, an array of defense mechanisms are activated, which include the generation of a complex signaling network, synthesis of antimicrobial compounds, lignification of cell walls, and expression of pathogenesis-related (PR) proteins or defense genes (Glazebrook, 2005). Frequently, lignins are frequently major structural components of secondary cell walls in vascular plants. They are not only associated with plant growth and development (Rinaldi et al., 2007; Thévenin et al., 2011; Anderson et al., 2015), but also with defense responses to environmental and biotic stresses (Nicholson and Hammerschmidt, 1992; Lange et al., 1995; Schenk et al., 2000; Cheong et al., 2002; Tronchet et al., 2010). Lignification has the potential to act in several ways in plant defense against pathogen infection. It can establish mechanical barriers to pathogen invasion, chemically modify cell walls to be more resistant to cell wall-degrading enzymes, increase the resistance of walls to the diffusion of toxins from the pathogens to the hosts and of nutrients from the hosts to the pathogens, produce toxic precursors and free radicals, and lignify and entrap the pathogens (Nicholson and Hammerschmidt, 1992; Bhuiyan et al., 2009). Unpolymerized monolignols may also have antimicrobial activities (Keen and Littlefield, 1979). However, genetic evidence of CAD function in plant disease resistance is very limited (Tronchet et al., 2010).

Angiosperm lignins are composed of three main subunits (referred to as monolignols) named the hydroxyphenyl (H), guaiacyl (G), and syringyl (S) monolignols. These monolignols are produced with three main branches and 11 enzymes, such as cinnamyl alcohol dehydrogenase (CAD), cinnamoyl CoA reductase (CCR), caffeic acid *O*-methyltransferase (COMT), and caffeoyl CoA 3-*O*-methyltransferase (CCOMT) (Anderson et al., 2015). CAD is a key enzyme in monolignol biosynthesis before oxidative polymerization in the cell wall (Baucher et al., 1996; Kim et al., 2004; Bhuiyan et al., 2009). In angiosperm plant species, CAD proteins have significant affinities for coniferyl aldehyde and sinapyl aldehyde (Brill et al., 1999). Numbers of CAD genes in the family have been provided by the complete genome sequencing and annotation of *Arabidopsis thaliana*, rice (*Oryza sativa*), and sorghum (*Sorghum bicolor*) (Sasaki and Sederoff, 2003; Tobias and Chow, 2005; Saballos et al., 2009). In *Arabidopsis*, nine CAD homologs have identified from the genome sequence database (Sibout et al., 2003), but only three (AtCAD5/CAD-D, AtCAD4/CAD-C, and AtCAD1) have been confirmed to be the important enzymes involved in monolignol biosynthesis (Kim et al., 2004; Sibout et al., 2005; Eudes et al., 2006). In *Arabidopsis*, through assessment on mutants (*cad*-*C cad*-*D*), the genetic and functional analyses indicate that the *CAD*-*C* (At3g19450) and *CAD*-*D* (At4g34230) are the primary genes being involved in lignin biosynthesis (Sibout et al., 2005), and act as essential components of defense response against virulent and avirulent strains of the bacterial pathogen *Pseudomonas syringae* pv. *tomato* (Tronchet et al., 2010). Recently, 12 and 14 genes belonging to CAD family have been identified in genomes of rice (Tobias and Chow, 2005) and sorghum (Saballos et al., 2009), respectively. The rice *OsCAD2* and sorghum *SbCAD2* have been shown to be responsible for lignin biosynthesis in rice (Zhang and Cheng, 2006) and sorghum (Saballos et al., 2009; Sattler et al., 2009), respectively. The rice *OsCAD7* mutant named flexible culm 1 displays a dramatic reduction in culm mechanical strength (Li et al., 2009). To date, through a genome-wide data mining in wheat EST database, 11 wheat CAD genes, namely *TaCAD1* to *TaCAD11*, have been identified (Ma, 2010). However, only one wheat CAD named TaCAD1 was functionally analyzed through RNA blot and biochemical assay. TaCAD1 has been shown to be a CAD enzyme in wheat stem, and might participate in lodging resistance (Ma, 2010). However, in common wheat, no genetic evidence for defense roles of CAD genes has yet been reported.

In this study, based on comparative transcriptomics between the sharp eyespot-resistant wheat line CI12633 and the susceptible wheat line Wenmai 6 following inoculation with *R. cerealis* R0301, the probe A_99_P016444, being homologous to certain plant CADs, was identified. Subsequently, this gene, named as *TaCAD12*, was cloned, and its defense role and the mechanism underlying the function were characterized through *TaCAD1*2-silencing and overexpressing wheat plants. The results of the CAD enzyme kinetic assay revealed that TaCAD12 protein possessed high catalytic efficiencies toward coniferyl aldehyde and sinapyl aldehyde. The functional dissection and preliminary mechanism analyses revealed that *TaCAD12* positively contributes to wheat resistance response against the sharp eyespot disease caused by *R. cerealis* through regulation of the expression of certain defense genes (*Defensin, PR10, PR17c*, and *Chitinase1*) and monolignol biosynthesis-related genes (*TaCAD1, TaCCR*, and *TaCOMT1*).

## Materials and Methods

### Plant and Fungal Materials

Six wheat (*T. aestivum*) lines/cultivars (cv.), including sharp eyespot-resistant lines CI12633 and Shanhongmai, moderately resistant line Niavt14, moderately susceptible lines Yangmai 158 and Yangmai 6, and susceptible line Wenmai 6, were used in this research.

A major Jiangsu virulent strain of the pathogen *R. cerealis* isolate R0301, was kindly provided by Profs. Huaigu Chen and Shibin Cai (Jiangsu Academy of Agricultural Sciences, China). A North China high-virulence strain of the pathogen *R. cerealis* isolate WK207 was provided by Prof. Jinfen Yu (Shandong Agricultural University, China).

### DNA or RNA Extraction and cDNA Synthesis

Genomic DNA for each sample was isolated from the wheat leaves using the CTAB method (Saghai-Maroof et al., 1984).

Total RNA was extracted using TRIzol (Invitrogen), and then subjected to Rnase-free Dnase I (Promega) digestion and purification.

The purified RNA sample (2 μg) was reverse-transcribed to cDNA using the FastQuant RT Kit with gDNase (Beijing Transgen Biotech, China).

### Isolation and Characterization of the TaCAD12 Sequence

The microarray analysis using the Agilent wheat microarray indicated that a probe A_99_P016444, corresponding to 3′-terminal sequence of a wheat cDNA sequence with accession number BT008979, was expressed at a significantly higher level in the sharp eyespot-resistant wheat CI12633 than in the susceptible wheat Wenmai 6. This gene cloned from stems of the resistant wheat CI12633, based on BT008979 sequence, was designated as *TaCAD12.* The 1393-bp full-length cDNA sequence of *TaCAD12* was amplified in 2 rounds of 3′-RACE from cDNA of CI12633 stems inoculated with *R. cerealis* R0301 for 4 d. The primers for the first round PCR were TaCAD12-U1 and AUAP; these for the second round PCR were TaCAD12-U2 and AUAP.

The deduced protein sequence was analyzed by Pfam database^1^ and smart software^2^ to predict conserved motifs. A phylogenetic tree was constructed using a neighbor-joining method implemented with MEGA 5.0 software.

### CAD Enzyme Kinetic Assay of TaCAD12 *In Vitro*

The ORF (open reading frame) sequence of *TaCAD12* was sub-cloned in frame to 3′-terminus of a GST (glutathione *S*-transferase) gene in the pGEX-4T-1 vector (GE Amersham), resulting in GST-TaCAD12 expression vector pGST-TaCAD12. The pGST-TaCAD12 DNA was transformed into competent cells of *Escherichia coli* (*E. coli*) strain BL21. The GST-TaCAD12 overexpression in the *E. coli* cells and the recombinant protein purification were conducted according to Dong et al. (2010).

The CAD enzyme activity of TaCAD12 was measured at OD_340_ according to the method of Goffner et al. (1992). Each reaction was monitored ten times with 1 min intervals. Following Ma (2010) and Saathoff et al. (2011), both *V*_max_ and *K*_m_ values were determined by extrapolation from the Lineweaver–Burk plots. The reactions were started by enzyme addition and terminated by holding at 85°C for 10 min. The final reaction mixture during the analysis of the reaction mechanism catalyzed by TaCAD12 contained 50 mM NADPH, 20 mM coniferyl aldehyde or sinapyl aldehyde, and 0.5 μg purified GST-TaCAD12 recombinant protein. Each reaction was prepared with two-component mixture and the third component were added later. Assay without NADPH was used as control.

### Virus-Induced Gene Silencing (VIGS) Assay for *TaCAD12* Function

In barley and wheat, the barley stripe mosaic virus (BSMV)-based VIGS (virus-induced gene silencing) assay has been shown to be an effective reverse genetic tool for rapidly investigating the functions of interest genes (Holzberg et al., 2002; Scofield et al., 2005; Zhou et al., 2007; Zhu et al., 2014). To generate the BSMV:TaCAD12 recombinant construct, a 190-bp sequence of *TaCAD12* (from 1011 to 1200 nucleotides in *TaCAD12* cDNA sequence) was sub-cloned in an antisense orientation into the *Nhe* I restriction site of the RNAγ of BSMV (**Supplementary Figure S2**). Following the protocols described by Holzberg et al. (2002) and Zhou et al. (2007), the tripartite cDNA chains of BSMV:TaCAD12 or the control BSMV:GFP virus genome were separately transcribed into RNAs, mixed, and used to inoculate the leaves of wheat CI12633 plants at the two-leaf stage. Then, the plants were grown in a 14 h light (25°C)/10 h dark (17°C) regime. To investigate if BSMV successfully infected CI12633 plants, and to test if *TaCAD12* transcript was down-regulated, at 8 days after the virus inoculation, the fourth leaves of the inoculated seedlings were collected and subjected to quantitative real-time PCR (Q-RT-PCR) to analyze the transcription of the BSMV coat protein (CP) gene and the transcription of *TaCAD12*. At 25 days after BSMV infection, the BSMV-infected CI12633 plants were further inoculated with *R. cerealis* isolate WK207 mycelia following Chen et al. (2008). They were scored at 10 and 40 dpi with *R. cerealis* WK207, respectively.

### *TaCAD12*-Overexpressing Construct and Transformation into Wheat

The *TaCAD12* ORF sequence with the *Spe* I and *Sac* I restriction sites was amplified with the primers TaCAD12-SPEI-U and TaCAD12-SACI-L and then sub-cloned into the *Spe* I and *Sac* I sites of a modified monocot transformation vector pAHC20-RSS1P (Christensen and Quail, 1996; Li et al., 2011) with a *c-myc* epitope tag. In the resulting overexpression transformation vector pRSS1P:myc-TaCAD12 (**Figure 5A**), the transcript of the *c-myc-TaCAD12* fusion gene is driven by a rice sucrose synthase-1 (*RSS1*) promoter (GenBank accession no. X64770.1) that was reported to be specifically expressing in the phloem tissue (Shi et al., 1994), and terminated by 3′-non-transcribed region of *Agrobacterium tumefaciens* nopaline synthase gene (*Tnos*). A total of 2,000 immature embryos of the wheat cultivar Yangmai 16 were transformed by biolistic bombardment using pRSS1P:myc-TaCAD12 plasmid DNA-contained golden powder following the protocol described by Chen et al. (2008).

### PCR and Western Blotting Analyses on *TaCAD12*-Overexpressing Wheat

The presence of the *TaCAD12-*overexpressing transgene in the transformed wheat plants was monitored by PCR using the specific primers, TaCAD12-4166-ZJF (located in *TaCAD12* coding sequence) and Tnos-3214L (located in *Tnos* of the transformation vector). PCR was performed in a 25 μl volume containing ∼200 ng genomic DNA, 12.5 μl 2x Taq MasterMix (Beijing ComWin Biotech Co. Ltd, China), 1 μl each primer (10 mM). The amplified product (277-bp size) specific to the introduced TaCAD12-Tnos chimera was resolved on a 1.2% agarose gel and visualized by ethidium bromide staining.

The c-myc-TaCAD12 fusion protein in the overexpressing wheat lines was visualized by Western blotting analysis. Total proteins were extracted from ∼0.5 g stems inoculated with *R. cerealis* R0301 for 40 days by using the tissue protein extraction kit (Beijing ComWin Biotech Co. Ltd, China). Total soluble proteins (∼10 μg) for each line were separated on 12% SDS-PAGE and transferred to polyvinyl difluoride membranes (Amersham). The blotting membranes were incubated with 2500-fold diluted Anti-c-myc Mouse Monoclonal Antibody (Beijing Transgen Biotech, China) at 4°C overnight, then incubated with 1500-fold diluted Goat Anti-Mouse IgG (H+L), HPR conjugated secondary antibody (Beijing Transgen Biotech, China) at 22-23°C for 1 h. The c-myc-TaCAD12 protein was visualized using the Pro-light HRP Chemiluminescent Kit (Beijing Transgen Biotech, China).

### CAD Kinetic Activity and Western Blot Analyses of TaCAD12 in Wheat Stems

Total proteins in *TaCAD12*-overexpressing and wild-type (WT, non-transgenic) wheat Yangmai 16 (recipient) lines were extracted from ∼0.5 g stems inoculated with *R. cerealis* R0301 for 40 days as mentioned above. The CAD enzyme kinetic activities in both *TaCAD12*-overexpressing and WT Yangmai 16 wheat lines were examined in the soluble protein fraction from their stem tissues according to the methods described by Goffner et al. (1992) and Ma (2010).

The Western blot analysis was performed to examine TaCAD12 protein in wheat stems with GST-TaCAD12 antibody. Briefly, the total proteins were extracted from stems of *TaCAD12*-overexpressing or WT Yangmai 16 wheat lines inoculated with *R. cerealis* R0301 for 40 days, then separated on 12% SDS-PAGE, and transferred to polyvinyl difluoride membranes (Amersham). The GST-TaCAD12 polyclonal antibody was raised in mouse injected by purified GST-TaCAD12 protein. The immunoblots were developed with the polyclonal GST-TaCAD12 antibody (1:1500), then 1500-fold diluted Goat Anti-Mouse IgG (H+L), HPR conjugated secondary antibody, and visualized using the Pro-light HRP Chemiluminescent Kit (Beijing Transgen Biotech, China).

### Assessment on Responses of Transgenic Wheat Plants to *R. cerealis*

At least 10 plants for each line of the *TaCAD12*-overexpressing wheat lines in T_1_–T_2_ generations and WT wheat Yangmai 16 were inoculated with sterilized grains harboring the well-developed mycelia of *R. cerealis* isolates WK207 or R0301 following the protocol of Wei et al. (2015). Based on the disease lesion squares, the infection type (IT) of each plant and disease index (DI) for each wheat line were categorized at harvest according to Cai et al. (2006).

### Analysis on Transcriptional Levels of Target Genes by Q-RT-PCR

Q-RT-PCR analysis with *TaCAD12* specific primers TaCAD12-Q-265F265F and TaCAD12-Q-557R was used to investigate the relative transcriptional levels of *TaCAD12* in various wheat plants. The tested wheat defense-marker genes include *Defensin* (NCBI accession no. CA630387), *PR10* (NCBI accession no. CA613496), *PR17c* (NCBI accession no. TA65181), and *chitinase1* (*Chit1*, NCBI accession no. CA665185). The tested wheat monolignol biosynthesis-related genes include *TaCAD1* (NCBI accession no. GU563724), *TaCCR* (NCBI accession no. DQ449508), and *TaCOMT1* (NCBI accession no. AY226581). Q-RT-PCR was performed using SYBR Green I Master Mix (TaKaRa) in a volume of 25 μl on an ABI 7300 RT-PCR system (Applied Biosystems). Reactions were set up using the following thermal cycling profile: 95°C for 15 min, followed by 41 cycles of 95°C for 10 s, 55°C for 20 s, and 72°C for 32 s. The relative transcriptional levels of the target genes was calculated using the 2^-ΔΔCT^ method (Livak and Schmittgen, 2001), where the wheat *Actin* gene *TaActin* was used as the internal reference. The relative transcriptional levels of the tested genes in the *TaCAD12*-overexpressing wheat lines or in BSMV:TaCAD12-infected wheat plants were relative to those in WT recipient or in BSMV:GFP-infected wheat plants.

### Primers

All primers used in this study are listed in **Supplementary Table S1**.

## Results

### *TaCAD12* Transcriptional Level Is Higher and More Enhanced by *R. cerealis* in the Resistant Wheat Lines

**FIGURE 1:**
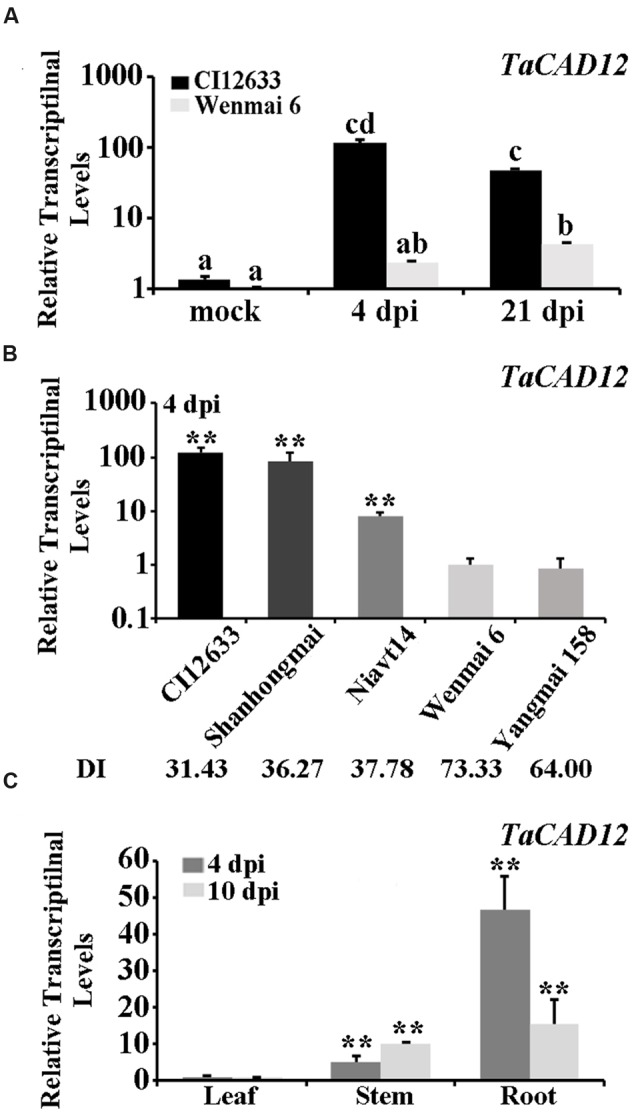
**Transcriptional patterns of *TaCAD12* in wheat stems after *R. cerealis* inoculation analyzed by Q-RT-PCR. (A)** The relative transcriptional levels of *TaCAD12* in the sharp eyespot-resistant wheat line CI12633 and the susceptible wheat line Wenmai 6 after inoculation with *R. cerealis* R0301 for 4 and 21 days or mock-treatment. The mock indicates an inoculation without *R. cerealis*. The transcriptional levels with different letters are significantly different from each other based on statistical comparisons (Student’s *t*-test, ^∗∗^*P* < 0.01). **(B)** The relative transcriptional levels of *TaCAD12* in sharp eyespot-resistant, moderately resistant, susceptible, and moderately susceptible wheat lines were measured after inoculation with *R. cerealis* R0301 for 4 days. The relative transcriptional levels of *TaCAD12* in different wheat lines were calculated relative to that of Wenmai 6. DI indicates the sharp eyespot disease index in each wheat line. **(C)** Transcription of *TaCAD12* in roots, stems and leaves of the wild-type (WT) Yangmai 16 at 4 and 10 dpi with *R. cerealis* R0301. Three technical replicates were averaged and statistically analyzed using Student’s *t*-test (^∗∗^*P* < 0.01). Bars indicate standard error of the mean (SEM).

Microarray-based comparative transcriptomic assay was used to identify defense-related genes of wheat in response to *R. cerealis* infection. Among the differentially expressed probes between sharp eyespot-resistant wheat line CI12633 and the susceptible wheat line Wenmai 6 at 4 and 21 day post inoculation (dpi) with *R. cerealis* R0301 (microarray raw data, GEO accession number GSE69245), the probe A_99_P016444, corresponding to 3′-terminal sequence of the wheat full insert mRNA sequence BT008979, showed 3.90-fold and 21.56-fold transcriptional increase in resistant wheat line CI12633 than in susceptible wheat line Wenmai 6 at 4 and 21 dpi with *R. cereali*s R0301, respectively. Blast search against the nucleotide acid sequence database in GenBank indicated that the sequence of BT008979 hit to *Lolium perenne CAD2* mRNA (AF472592.1, sharing 89% identity), and *Brachypodium distachyon* putative *CAD5* mRNA (XM_003573500.2, 88% identity). Further sequence alignment showed that the sequence of BT008979 is homologous to the previously reported wheat *TaCAD3* gene (TC143265, 89.11% identity), but less than 49.11% identity with *TaCAD1*-*TaCAD2* and *TaCAD4*-*TaCAD11* (Ma, 2010). Thus, this gene, which was cloned from the resistant wheat line CI12633 based on BT008979, was designated as *TaCAD12*. Q-RT-PCR analysis results showed that transcriptional level of *TaCAD12* was higher in the resistant wheat line CI12633 stems than in susceptible wheat line Wenmai 6 stems with *R. cerealis* R0301 inoculation or mock-inoculation without *R. cerealis* (**Figure 1A**). The Q-RT-PCR results were in agreement with the microarray analysis trend. The transcriptional level of *TaCAD12* was markedly increased in the resistant wheat line CI12633 after *R. cerealis* R0301 inoculation for 4 and 21 days, whereas the transcriptional induction in susceptible wheat line Wenmai 6 was relatively weaker (**Figure 1A**). Moreover, following *R. cerealis* R0301 inoculation, *TaCAD12* transcriptional levels were significantly higher in three sharp eyespot-resistant wheat lines (CI12633, Shanghongmai, and Niavt14) than in two susceptible lines (Wenmai 6 and Yangmai 158) (**Figure 1B**). Additionally, the tissue expression analysis showed that the transcriptional level of *TaCAD12* was the highest in roots, intermediate in stems, and the lowest in the leaves of the wheat line Yangmai 16 at both 4 and 10 dpi with *R. cerealis* R0301 (**Figure 1C**). These results suggested that *TaCAD12* might participate in wheat defense response to *R. cerealis* infection.

### *TaCAD12* Encodes a Cinnamyl Alcohol Dehydrogenase

**FIGURE 2:**
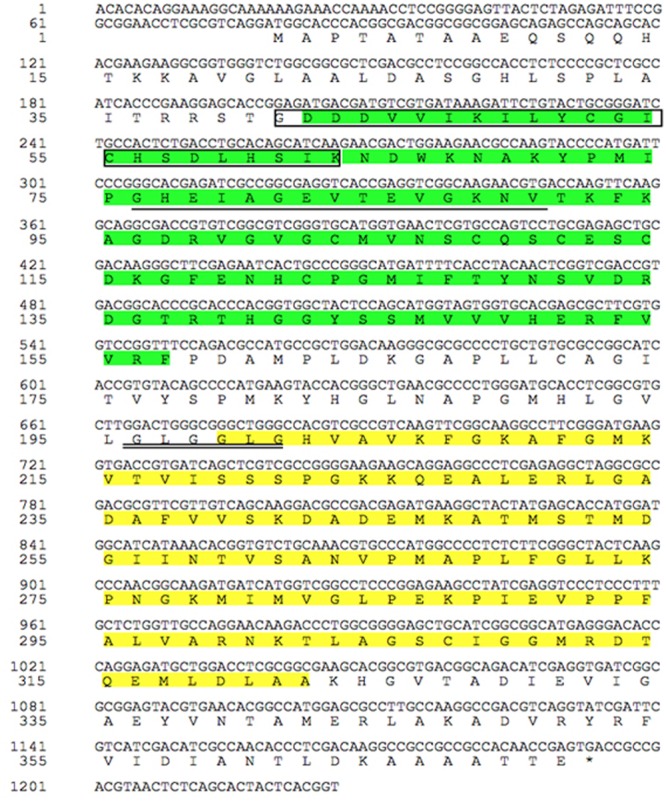
**The analyses of nucleotide sequence and deduced amino acid sequence of TaCAD12.** The green part represents alcohol dehydrogenase domain (42–157 aa), in which the amino acid sequence in box represents the first Zn binding site in TaCAD12, and the amino acid sequence with single underline is the second Zn binding site. The Zinc-binding dehydrogenase domain (199–323 aa) is marked by yellow spaces, in which the highly conserved motif in CAD is marked with double underlines (196–201 aa).

The full-length cDNA sequence of *TaCAD12* was cloned from cDNA of CI12633 stems inoculated with *R. cerealis* R0301, and was deposited in GenBank with the accession no. KX585233. The *TaCAD12* cDNA sequence with 1393-bp contains an ORF with 1116-bp (from 79 to 1194 nt), the 5′-untranslated region (UTR) with 78-bp, and 3′-UTR with 200-bp (**Figure 2**). The *TaCAD12* cDNA sequence shares an 87.53% identity with the previously reported wheat *TaCAD3* gene (TC143265). The predicted protein of TaCAD12 is consisted of 371 amino acids with a molecular weight of 39.43 kDa and a theoretical *iso*-electric point of 7.39. As shown in **Figure 2**, the TaCAD12 protein contains one alcohol dehydrogenase domain (42–157 aa) with two Zn binding sites (41–63 and 76–90 aa) and one Zinc-binding dehydrogenase domain (199–323 aa) with one highly conserved motif (^196^GLGGLG^201^) in CAD. The motif GLGGLG has been shown to participate in binding the pyrophosphate group of NADP^+^ (Youn et al., 2006).

**FIGURE 3:**
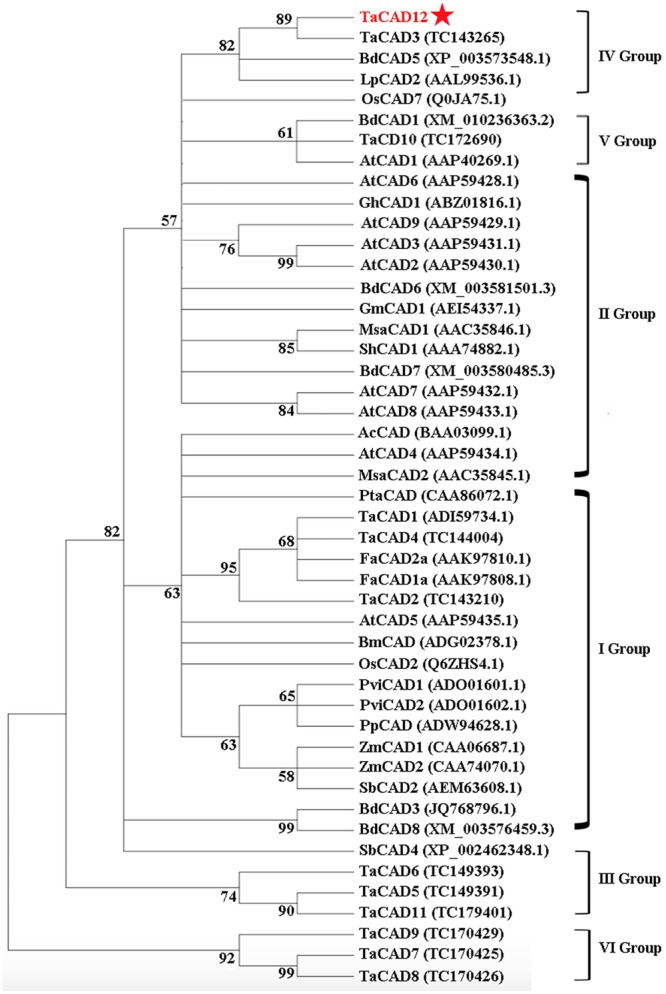
**Phylogenetic relationships of TaCAD12 and 46 other CAD proteins in plants.** The phylogenetic tree was constructed by using neighbor-joining phylogeny of MEGA 5.0 following ClustalW method, and is showed in bootstrapped manner. The GenBank accession number or TIGR number of each protein was marked in the bracket behind the corresponding proteins.

Following the classification method announced by Saballos et al. (2009), a phylogenetic tree, including 12 wheat CAD proteins and 35 CADs from other plant species, was constructed and divided into six groups (**Figure 3**). The proteins TaCAD12, TaCAD3, BdCAD5, LpCAD2, and OsCAD7 belonged to IV Group of CAD family (**Figure 3**). The protein sequence alignment indicated that the protein sequence of TaCAD12 shares 83.79, 88.44, 76.16, and 67.62% identities with TaCAD3, BdCAD5, LpCAD2, and OsCAD7, respectively. The functions of TaCAD3, BdCAD5, and LpCAD2 have not been studied yet. The OsCAD7 is confirmed to have strong activity toward coniferyl aldehyde and weak activity toward sinapyl aldehyde, and controls culm mechanical strength in rice (Li et al., 2009). Additionally, the protein sequence of TaCAD12 shares 47.81 and 47.39% identities with PviCAD1 and PviCAD2, two reported CADs in switchgrass (*Panicum Virgatum* L.), respectively (Saathoff et al., 2011, 2012). These results suggested that TaCAD12 encoded a CAD.

### TaCAD12 Possesses Catalytic Activities toward Coniferyl Aldehyde and Sinapyl Aldehyde

To investigate if TaCAD12 has activities toward coniferyl aldehyde and sinapyl aldehyde, we analyzed the kinetics of recombinant GST-TaCAD12 protein toward two putative substrates (coniferyl aldehyde and sinapyl aldehyde). The catalytic properties of TaCAD12 were labeled by *V*_max_ and *K*_m_ values. The *V*_max_ values of the GST-TaCAD12 protein toward coniferyl aldehyde and sinapyl aldehyde are 347.58 ± 17.38 and 315.01 ± 18.58 nkat mg^-1^ protein, respectively. These results indicated that TaCAD12 protein showed high catalytic efficiencies toward both coniferyl aldehyde and sinaphy aldehyde, even though a slightly higher efficiency was displayed for catalyzing coniferyl aldehyde. Meanwhile, the *K*_m_ values of the GST-TaCAD12 protein toward coniferyl aldehyde and sinapyl aldehyde were 28.91 ± 3.67 and 34.18 ± 5.56 μM, respectively. Additionally, as shown in **Supplementary Figure S1**, the catalytic reactions of GST-TaCAD12 protein toward both coniferyl aldehyde and sinapyl aldehyde happened immediately, regardless of the component adding order. These results prove that TaCAD12 is an authentic CAD enzyme, and possesses catalytic activities toward both coniferyl aldehyde and sinapyl aldehyde.

### Down-Regulation of *TaCAD12* Represses Wheat Resistance to Sharp Eyespot

**FIGURE 4:**
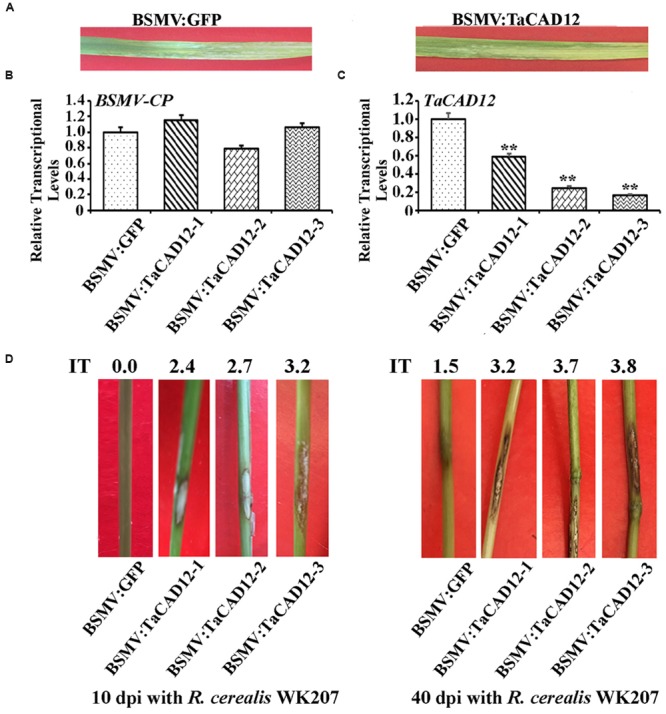
**Molecular characterizations and *R. cerealis* responses of *TaCAD12*-silencing CI12633 plants. (A)** BSMV symptoms on the leaves of BSMV:GFP- and BSMV:TaCAD12-infected wheat CI12633 plants. **(B)** Transcriptional levels of BSMV coat protein (*CP*) gene in the leaves of the BSMV:GFP- and BSMV:TaCAD12-infected wheat CI12633 plants at 8 days post virus inoculation. **(C)** Transcriptional levels of *TaCAD12* in the leaves of the BSMV:GFP- and BSMV:TaCAD12-infected wheat CI12633 plants at 8 days post virus inoculation. Statistically significant differences between BSMV:TaCAD12-inoculated plants and BSMV:GFP-inoculated plants at the same time point based on three replications using Student’s *t*-test (^∗∗^*P* < 0.01). **(D)** Sharp eyespot symptoms of BSMV:GFP- and BSMV:TaCAD12-infected wheat CI12633 plants following *R. cerealis* WK207 inoculation for 10 and 40 days. IT indicates sharp eyespot infection type in each wheat line.

When the sharp eyespot-resistant wheat CI12633 plants had been infected with BSMV for 8 days, BSMV-infected symptom was present in both BSMV:GFP- and BSMV:TaCAD12-inoculated CI12633 plants (**Figure 4A**), and the expression of BSMV *CP* gene could be detected from stems of these plants (**Figure 4B**). The results indicated that these BSMV:GFP and BSMV:TaCAD12 viruses successfully infected these viruses-inoculated wheat plants. The *TaCAD12* transcriptional level was substantially reduced in BSMV:TaCAD12-infected CI12633 plants, indicating that *TaCAD1*2 was successfully knock-downed in BSMV:TaCAD12-infected CI12633 plants (**Figure 4C**). Then, these plants were further inoculated with *R. cerealis* isolate WK207 to evaluate the defense role of *TaCAD12*. At 10 dpi with *R. cerealis* WK207, the sharp eyespot symptom (brown lesion) was present on the fungal inoculated sheaths and stems of BSMV:TaCAD12-infected CI12633 plants, but absent on the fungal inoculated stems of BSMV:GFP-treated CI12633 plants (**Figure 4D**). At 40 dpi with *R. cerealis* WK207, more serious symptoms of sharp eyespot were present on the stems of BSMV:TaCAD12-infected CI12633 plants (average IT: 3.33), compared with those on the stems of BSMV:GFP-treated CI12633 plants (average IT: 1.46) (**Figure 4D**). These results indicated that the down-regulation of *TaCAD12* transcript in CI12633 impaired host resistance to sharp eyespot caused by *R. cerealis*, and that *TaCAD12* seems to be essential to the wheat resistance against *R. cerealis* infection.

### Overexpression of *TaCAD12* Increases Wheat Resistance to Sharp Eyespot

**FIGURE 5:**
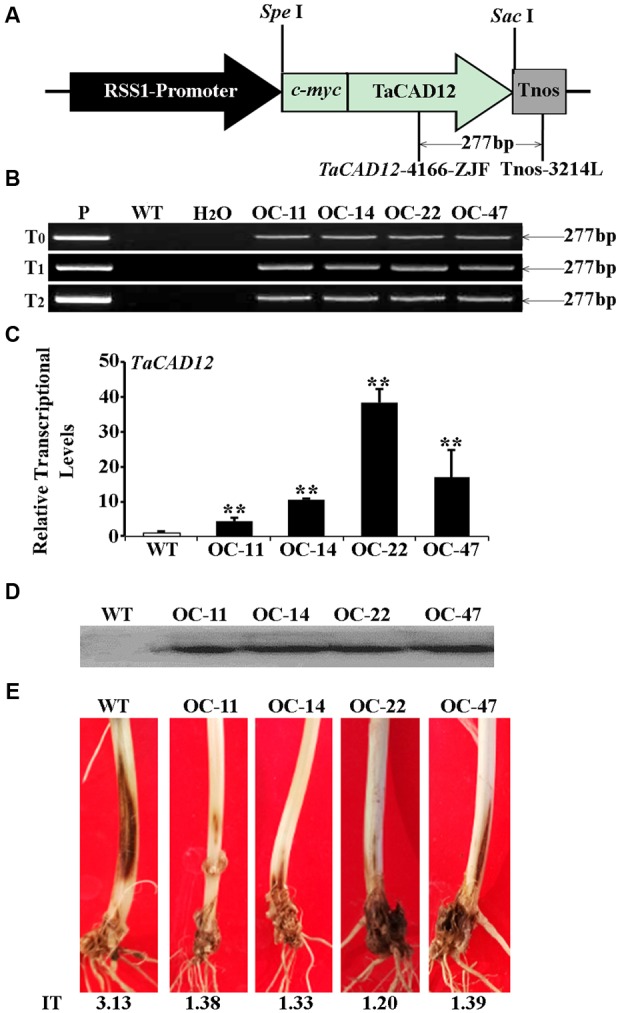
**Molecular characterizations and *R. cerealis* responses of *TaCAD12*-overexpressing wheat lines. (A)** Schema of the transformation vector pRSS1P:myc-TaCAD12. *TaCAD12* in the transformation vector was driven by the rice sucrose synthase-1 promoter (RSS1P) and terminated by *Tnos*. The arrows region indicates the 277-bp fragment amplified in PCR assays using transgene-specific primers (TaCAD12-4166-ZJF/Tnos-3214L). **(B)** PCR patterns of *TaCAD12-*overexpressing transgenic lines in T_0_–T_2_ generations and WT (non-transgenic) Yangmai 16 using transgene-specific primers. P: transformed vector pRSS1P:myc-TaCAD12, WT: WT Yangmai 16, M: 100-bp DNA ladder. **(C)** The transcriptional levels of *TaCAD12* in the stems of T_2_ *TaCAD12-*overexpressing lines and WT Yangmai 16 through Q-RT-PCR analysis. The transcriptional levels in the transgenic lines (OC-11, OC-14, OC-22, and OC-47) were quantified relative to that in WT Yangmai 16. **(D)** Western blot pattern in stems of these four *TaCAD12*-overexpressing lines and WT Yangmai 16 was probed with an anti-c-myc antibody. **(E)** Sharp eyespot symptoms of the *TaCAD12*-overexpressing lines and WT Yangmai 16 in16 in T_2_ generation after the 40 dpi with *R. cerealis* R0301. IT indicates sharp eyespot infection type in each wheat line.

The role of *TaCAD12* in resistance response to *R. cerealis* was further studied by developing and evaluating *TaCAD12*-overexpressing wheat lines. By using the primers specific to the overexpression transformation vector pRSS1P:myc-TaCAD12 (**Figure 5A**), PCR analysis results showed that the introduced *TaCAD12* transgene was present in four wheat lines (OC-11, OC-14, OC-22, and OC-47) in T_0_–T_2_ generations (**Figure 5B**), suggesting that the transgene could be inheritable in these four lines. Q-RT-PCR analyses indicated that the transcriptional levels of *TaCAD12* in these four transgenic lines (OC-11, OC-14, OC-22, and OC-47) were markedly elevated than in WT Yangmai 16 (recipient) (**Figure 5C**), and the introduced *TaCAD12* was overexpressed in these four lines. The Western blotting results proved that the introduced *myc-TaCAD12* gene was translated into the fusion protein in these four overexpressing lines, but not in WT Yangmai 16 (**Figure 5D**). The sharp eyespot severity assessments in two successive (T_1_–T_2_) generations showed that compared with WT Yangmai 16, all these four *TaCAD12-*overexpressing wheat lines (OC-11, OC-14, OC-22, and OC-47) displayed significantly enhanced resistance to sharp eyespot caused by both *R. cerealis* R0301 and *R. cerealis* WK207 (**Table 1**; **Figure 5E**). For example, the ITs and DIs of these four *TaCAD12*-overexpressing lines in T_2_ generation were 1.20–1.39 and 24.00–27.80, respectively, whereas the average IT and DI of WT Yangmai 16 were 3.13 and 62.60, respectively (**Table 1**). These results revealed that *TaCAD12* positively contributed to wheat resistance against sharp eyespot caused by *R. cerealis* infection.

**Table 1:** *R. cerealis* responses of *TaCAD12*-overexpression lines and WT wheat Yangmai 16.

Lines	T_1_		T_2_	
	Infection Type	Disease Index	Infection Type	Disease Index
OC-11	1.25^∗∗^	25.00^∗∗^	1.38^∗∗^	27.60^∗∗^
OC-14	1.00^∗∗^	20.00^∗∗^	1.33^∗∗^	26.60^∗∗^
OC-22	1.50^∗∗^	30.00^∗∗^	1.20^∗∗^	24.00^∗∗^
OC-47	1.50^∗∗^	30.00^∗∗^	1.39^∗∗^	27.80^∗∗^
WT	2.67	53.48	3.13	62.60

*OC-11, OC-14, OC-22, and OC-47 indicate *TaCAD12*-overexpressing transgenic wheat lines and WT indicates non-transgenic wheat Yangmai 16 (recipient). The Statistically significant differences in the infection types and disease indexes between *TaCAD12*-overexpression wheat lines and WT wheat Yangmai 16 were determined based on the three biological replications using Student’s *t*-test (^∗∗^*P* < 0.01, statistically significant difference). The wheat plants in T_*1*_ and T_*2*_ generations were inoculated with *R. cerealis* isolate WK207 and *R. cerealis* isolate R0301, respectively.*

**FIGURE 6:**
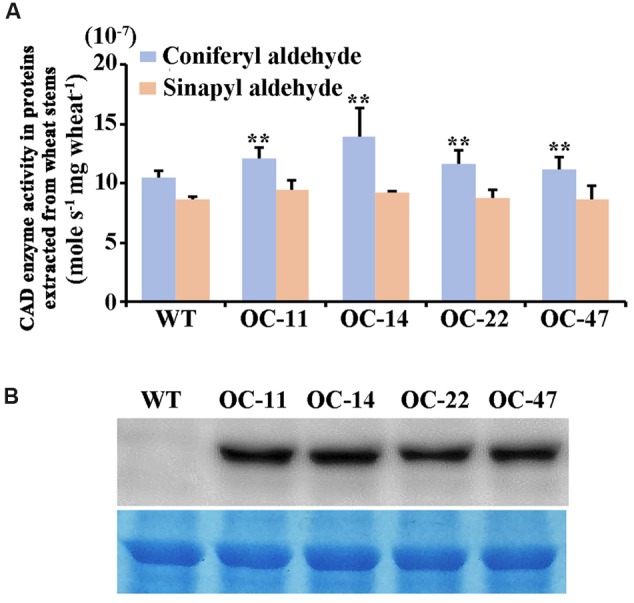
**CAD kinetic activity and Western blot analyses of TaCAD12 in wheat stems. (A)** Comparison of catalytic activities (mole s^-1^ mg wheat^-1^) toward both coniferyl aldehyde and sinapyl aldehyde in *TaCAD12*-overexpressing and non-transgenic Yangmai 16 wheat lines. Proteins were extracted from the stem tissues of *TaCAD12*-overexpressing and WT Yangmai 16 wheat lines after inoculated with *R. cerealis* R0301 for 40 d. Statistically significant differences between *TaCAD12*-overexpressing lines and WT Yangmai 16 were determined based on three replications using Student’s *t*-test (^∗∗^*P* < 0.01). **(B)** Western blot analysis of TaCAD12 protein from stem tissues of *TaCAD12*-overexpressing and WT Yangmai 16 wheat lines after 40 dpi with *R. cerealis* R0301. The blot was probed with the polyclonal GST-TACAD12 antibody (upper panel), and equal loading of protein samples was shown by coomassie brilliant blue staining (lower panel).

Furthermore, we examined the enzyme catalytic activities toward coniferyl aldehyde and sinapyl aldehyde (**Figure 6A**) and the TaCAD12 protein levels (**Figure 6B**) in the stem tissues of both *TaCAD12*-overexpressing and WT Yangmai 16 wheat lines. The enzyme catalytic activity toward coniferyl aldehyde in these four *TaCAD12*-overexpressing wheat lines (OC-11, OC-14, OC-22, and OC-47) was significantly higher than in WT Yangmai 16 (**Figure 6A**). The catalytic activity toward sinapyl aldehyde was slightly higher in these four *TaCAD12*-overexpressing wheat lines (OC-11, OC-14, OC-22, and OC-47) than in WT wheat Yangmai 16 (**Figure 6A**), although the difference was not significant. Western blotting analysis showed that with the polyclonal GST-TACAD12 antibody, a single band was detected only in the stem tissues of these four *TaCAD12*-overexpressing wheat lines (OC-11, OC-14, OC-22, and OC-47), but not in WT Yangmai 16 (**Figure 6B**). These results indicated that compared with WT Yangmai 16, these four *TaCAD12*-overexpressing wheat lines (OC-11, OC-14, OC-22, and OC-47) possessed both higher TaCAD12 protein levels and higher catalytic efficiency of CAD.

### *TaCAD12* Positively Regulates the Expression of Certain Defense- and Monolignol Biosynthesis-Related Genes

**FIGURE 7:**
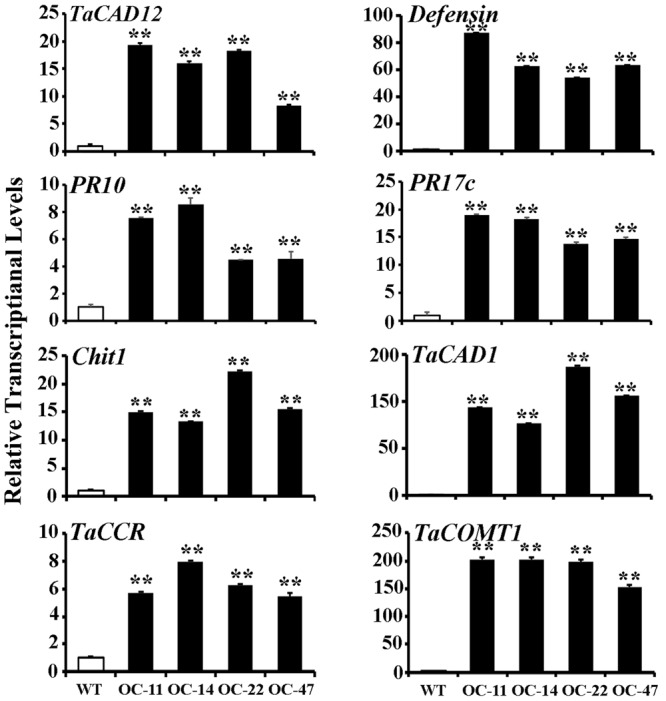
**Transcriptional levels of *TaCAD12*, defense, and monolignol biosynthesis-related genes in *TaCAD12*-overexpressing and WT Yangmai 16 wheat lines after *R. cerealis* inoculation.** Relative transcriptional abundances of the tested genes (*TaCAD12, Defensin, PR10, PR17c, Chit1, TaCAD1, TaCCR*, and *TaCOMT1*) in *TaCAD12*-overexpressing wheat lines (OC-11, OC-14, OC-22, and OC-47) were quantified relative to that in WT Yangmai 16 at 40 dpi with *R. cerealis* R0301. Statistically significant differences between *TaCAD12*-overexpressing lines and WT Yangmai 16 were determined based on three replications using Student’s *t*-test (^∗∗^*P* < 0.01).

**FIGURE 8:**
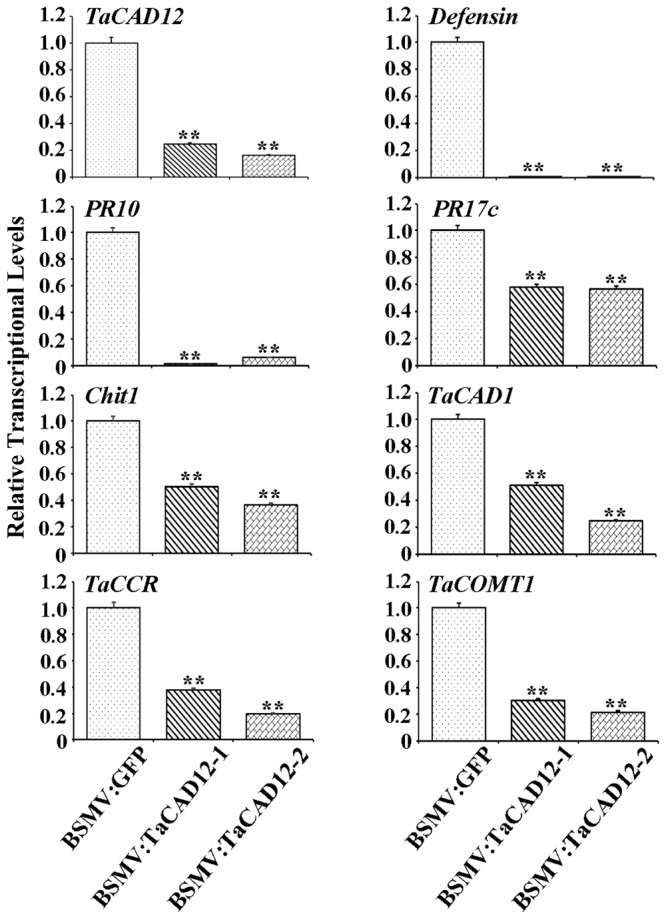
**The transcriptional levels of *TaCAD12*, defense, and monolignol biosynthesis-related genes in BSMV:TaCAD12- and BSMV:GFP-infected CI12633 plants after *R. cerealis* inoculation.** Relative transcriptional abundances of the tested genes (*TaCAD12, Defensin, PR10, PR17c, Chit1, TaCAD1, TaCCR*, and *TaCOMT1*) in BSMV:TaCAD12-infected CI12633 plants were quantified relative to that in BSMV:GFP-infected CI12633 plants after *R. cerealis* WK207 inoculation for 40 days. Statistically significant differences between BSMV:TaCAD12- and BSMV:GFP- inoculated CI12633 plants were determined based on three replications using Student’s *t*-test (^∗∗^*P* < 0.01).

In plants, defense genes play important roles in the resistance to pathogens. The monolignol biosynthesis-related genes (*CAD, CCR*, and *COMT*) may also participate in pathogen-resistance (Bhuiyan et al., 2009; Tronchet et al., 2010). To explore if *TaCAD12* regulates defense and monolignol biosynthesis-related genes in the resistance response to *R. cerealis* infection, we investigated the transcription changes of certain defense and monolignol biosynthesis-related genes (*Defensin, PR10, PR17c, chitinase1, TaCAD1, TaCCR*, and *TaCOMT1*) in *TaCAD12-*overexpressing wheat plants and *TaCAD12-*silencing wheat plants as well as their control plants. After 40 dpi with *R. cerealis* R0301, the transcriptional levels of these tested genes were significantly increased in enhanced-resistant *TaCAD12-*overexpressing wheat lines than in the susceptible WT Yangmai 16 plants (**Figure 7**), suggesting that the overexpression of *TaCAD12* up-regulated the transcriptional levels of these defense and monolignol biosynthesis-related genes. On the contrary, the transcriptional levels of these tested genes were significantly decreased in reduced-resistant *TaCAD12-*silencing wheat plants than in the BSMV:GFP-infected CI12633 plants after inoculation with *R. cerealis* WK207 for 40 days (**Figure 8**). These results indicated that *TaCAD12* positively regulated the expression of these defense and monolignol biosynthesis-related genes in wheat.

## Discussion

In this study, the wheat CAD gene *TaCAD12* was identified based on comparative transcriptomics between sharp eyespot-resistant wheat line CI12633 and susceptible wheat line Wenmai 6. Comparing with susceptible wheat lines, the transcriptional level of *TaCAD12* gene was higher and could be significantly enhanced in the sharp eyespot-resistant wheat lines (CI12633 and Shanhongmai) after *R. cerealis* infection. In *Arabidopsis*, the expression of *CAD-D* was obviously induced after inoculation with *Pseudomonas syringae* pv. *tomato*; two CAD proteins CAD-C and CAD-D not only act as key enzymes in lignin biosynthesis (Sibout et al., 2005), but also play essential roles in plant defense against infection of the bacterial (*Pseudomonas syringae* pv. *tomato*) pathogen (biotrophic pathogen, Tronchet et al., 2010). Additionally, the transcriptional level of *TaCAD12* was the highest in roots that are the original infecting site of *R. cerealis*, and intermediate in stems that are the main occurring site of sharp eyespot symptom. These results imply that *TaCAD12* may participate in defense response of wheat to *R. cerealis* infection. Importantly, silencing of *TaCAD12* in the resistant wheat line CI12633 significantly impairs host resistance to *R. cerealis, TaCAD12*-overexpressing wheat lines displayed significantly increased resistance during whole growth stages. These results clearly reveal that *TaCAD12* positively contributes to resistance against *R. cerealis* infection in wheat. To our knowledge, this is the first report about a CAD member positively participating in plant resistance responses to necrotrophic fungal pathogens through both partial loss-of-function (gene-silencing) analysis and partial gain-of-function (overexpressing transgene) analysis. To date, the mechanisms of plant responses to necrotrophic pathogens have been limited. Our results extend the current knowledge of plant defenses against pathogens.

The protein sequence and phylogenetic tree analyses suggest that TaCAD12 is closer to TaCAD3, than to BdCAD5, LpCAD2, and OsCAD7 in the same Group (IV), but far from the wheat TaCAD1 in I Group of CAD family. In IV Group, only OsCAD7 has been reported to have strong activity toward coniferyl aldehyde and weak activity toward sinapyl aldehyde, which influences lignin contents and mechanical strength of rice culm (Li et al., 2009). The results of CAD activity analysis *in vitro* prove that TaCAD12 protein is an authentic CAD enzyme, similar to the reported TaCAD1. TaCAD1 may be involved in monolignol biosynthesis and lodging resistance in wheat (Ma, 2010). TaCAD12 possesses catalytic activities toward both coniferyl aldehyde and sinapyl aldehyde. Moreover, our analysis results of CAD catalytic activity and TaCAD12 protein levels in wheat stems suggest that higher TaCAD12 expression levels could lead to relatively stronger CAD kinetic activities in *TaCAD12*-overexpressing wheat lines (OC-11, OC-14, OC-22, and OC-47) compared with WT Yangmai 16. The CAD catalytic activities play a key role in biosynthesis of monolignols and lignin abundance, and the mechanical properties of plants (Mellerowice et al., 2001; Sibout et al., 2003, 2005; Bhuiyan et al., 2009; Sattler et al., 2009; Li et al., 2009; Anderson et al., 2015).

The elevated expression levels of certain monolignol biosynthesis-related enzymes have been documented to occur during plant-microbe interaction (Nicholson and Hammerschmidt, 1992; Bhuiyan et al., 2009; Tronchet et al., 2010). Mutations or down-regulation of monolignol biosynthesis-related genes lead to reduction in lignin biosynthesis/concentration (Piquemal et al., 1998; Sibout et al., 2005; Do et al., 2007; Sattler et al., 2009; Tamasloukht et al., 2011; Thévenin et al., 2011; Scully et al., 2016). For example, in sorghum, *brown midrib 6* mutants (mutations in the evolutionary conserved amino acids of *SbCAD2*) have been shown to have limited CAD activity and to reduce the abundance of lignin (Sattler et al., 2009; Scully et al., 2016). Importantly, the interruption of monolignol biosynthesis through CAD and COMT inhibitors or the gene-silencing of monolignol biosynthesis-related enzymes could increase the susceptibility of barley and diploid wheat to the pathogen *Blumeria graminis* (a biotrophic fungal pathogen), indicating that monolignol biosynthesis is critically important for host defense against biotrophic pathogen invasion (Kruger et al., 2002; Bhuiyan et al., 2009). In our study, following *R. cerealis* infection, comparing with their control wheat plants, the transcriptional levels of monolignol biosynthesis-related genes (*TaCAD12, TaCAD1, TaCCR*, and *TaCOMT1*) were reduced in more susceptible *TaCAD12*-silenced wheat plants, but were increased in *TaCAD12*-overexpressing wheat lines with enhanced-resistance to sharp eyespot. The results indicated that *TaCAD12* positively regulated the expression of *TaCAD1, TaCCR*, and *TaCOMT1*, and might enhance monolignol biosynthesis, resulting in increased-resistance to sharp eyespot in wheat. These data suggest that TaCAD12 positively regulates host resistance to sharp eyespot possibly through elevating monolignol biosynthesis.

Defense genes played a vital role in plant resistance against pathogen infections. In *Arabidopsis, cad-C*/*cad-D* mutations negatively affected the expression of *PR1* and *PR5* (defense) genes after inoculation with virulent strain of *P. syringae* pv. *tomato* (Tronchet et al., 2010). In order to explore if *TaCAD12* regulates defense genes in wheat resistance response to *R. cerealis*, we investigated the transcriptional levels of four wheat defense marker genes, including *Defensin, PR10, PR17*c, and *Chit1*, in *TaCAD12*-overexpressing and silencing wheat plants as well as their control plants. The results showed that transcriptional levels of *Defensin, PR10, PR17*c, and *Chit1* in stems of *TaCAD12*-overexpression wheat plants were elevated than in WT wheat plants, whereas these genes exhibited transcriptional reduction in *TaCAD12*-silencing wheat plants compared with the control plants. These data suggested that *TaCAD12* could up-regulate the transcription of these four defense genes tested, consequently leading to the increased resistance against *R. cerealis* infection.

## Conclusion

The wheat CAD gene *TaCAD12* was identified via comparative transcriptomics. The transcriptional levels of *TaCAD12* are higher and markedly elevated after the infection of *R. cerealis* in resistant wheat lines. The TaCAD12 protein is an active CAD enzyme with catalytic activities toward both coniferyl aldehyde and sinapyl aldehyde. TaCAD12, acting as a positive contributor, appears to be essential to resistance response to *R. cerealis* infection through regulating the expression of certain defense genes and monolignol biosynthesis-related genes in wheat. This study provides insights into the roles of CAD members in plant defense responses. *TaCAD12* is a candidate gene to improve wheat resistance to sharp eyespot.

## Author Contributions

ZZ designed the research and wrote the paper. WR, ML, and TS performed the cloning, sequencing, enzyme kinetic and transcriptional analyses, and functional assays. XW analyzed the Q-RT-PCR data. HX and LD conducted wheat transformation.

## Conflict of Interest Statement

The authors declare that the research was conducted in the absence of any commercial or financial relationships that could be construed as a potential conflict of interest.

## Footnotes

1 http://pfam.xfam.org

2 http://smart.embl-heidelberg.de/

## Supplementary Material

Figure S1 **The reaction patterns catalyzed by the GST-TaCAD12 recombinant protein.** The reaction patterns catalyzed by TaCAD12 with (A) coniferyl aldehyde and (B) sinapyl aldehyde were measured under different mixture component adding orders. The third component added was (i) purified GST-TaCAD12 recombinant protein, (ii) NADPH, and (iii) substrates (coniferyl aldehyde and sinapyl aldehyde), respectively.

Figure S2 **Schematic representation of the *barley strip mosaic virus (BSMV)* genome organization and BSMV recombinant constructs engineered to express GFP and *TaCAD12* fragments as inverted repeats.** RNAs α, β, and γ were as described (Holzberg et al., 2002). The 190-bp *TaCAD12* fragment (from 1011 to 1200 nt in TaCAD12 cDNA sequence) was sub-cloned in an antisense orientation into the *Nhe* I restriction site of the RNA γ of BSMV.

Table S1 **Sequences of primers used in this study**.
