# Supplementary material for: A Wheat Cinnamyl Alcohol Dehydrogenase TaCAD12 Contributes to Host Resistance to the Sharp Eyespot Disease
Source: Front Plant Sci. 2016 Nov 16;7:1723. doi: 10.3389/fpls.2016.01723 (PMC5110560; doi:10.3389/fpls.2016.01723)
Supplement: Figure S1 — The reaction patterns catalyzed by the GST-TaCAD12 recombinant protein. The reaction patterns catalyzed by TaCAD12 with (A) coniferyl aldehyde and (B) sinapyl aldehyde were measured under different mixture component adding orders. The third component added was (i) purified GST-TaCAD12 recombinant protein, (ii) NADPH, and (iii) substrates (coniferyl aldehyde and sinapyl aldehyde), respectively. [file Table_1.DOCX]

**Supporting Information Tables S1**

**Table S1** Sequences of primers used in this study

| Primer Name | Sequence (5'-3') | Usage |
| --- | --- | --- |
| TaCAD12-U1 | 5’-TCGCCTCACACACAGGAAAG-3’ | PCR for *TaCAD12* amplification |
| TaCAD12-U2 | 5’-ACACACAGGAAAGGCAAAAA-3’ |  |
| AUAP | 5'-GGCCACGCGTCGACTAGTAC-3' |  |
| TaCAD12-PG-F | 5’- AATGGATCCATGGCACCCACGGCGACG-3’ | GST-TaCAD12 recombinant vector |
| TaCAD12-PG-R | 5’-AGTGAATTCTCACTCGGTTGTGGCGGC-3’ |  |
| BSMV-CPF | 5'-TGACTGCTAAGGGTGGAGGA-3' | Q-RT-PCR wheat BMSV coat protein |
| BSMV-CPR | 5'-CGGTTGAACATCACGAAGAGT- 3' |  |
| TaCAD12-SPEi-U | 5'-GAACTAGT ATGGCACCCACGGCGACG-3' | TaCAD12-overexpressing transformation vector |
| TaCAD12-SACi-L | 5'-CAGAGCTCTCACTCGGTTGTGGCGGC-3' |  |
| TaCAD12-Q-265F | 5’-AACGTGACCAAGTTCAAGGC-3’ | Q-RT-PCR wheat *TaCAD12* transcript |
| TaCAD12-Q-557R | 5'-TTCAGCCCGTGGTACTTCAT-3' |  |
| Defensin-Q-F | 5'-ATGTCCGTGCCTTTTGCTA-3' | Q-RT-PCR wheat *Defensin* transcript |
| Defensin-Q-R | 5'-CCAAACTACCGAGTCCCCG-3' |  |
| PR10-Q-F | 5'-CGTGGAGGTAAACGATGAG-3’ | Q-RT-PCR wheat *PR10* transcript |
| PR10-Q-R | 5'-GCTAAGTGTCCGGGGTAAT-3' |  |
| PR17c-Q-F | 5'-ACGACATCACGGCGAGGT-3’ | Q-RT-PCR wheat *PR17c* transcript |
| PR17c-Q-R | 5'-CACGGGGAAAGAGAGGATGA-3' |  |
| Chit1-Q-F | 5'-ATGCTCTGGGACCGATACTT-3' | Q-RT-PCR wheat C*hitinase1* transcript |
| Chit1-Q-R | 5'-AGCCTCACTTTGTTCTCGTTTG-3' |  |
| TaCAD1-Q-F | 5'-AAAAGCAGAGGGAGAAAGAGC-3' | Q-RT-PCR wheat *TaCAD1* transcript |
| TaCAD1-Q-R | 5'-AACGGAAACAGGTCACAAACAT-3' |  |
| TACCR-Q-F | 5'-CGTGGCATCTCCATCTCA-3' | Q-RT-PCR wheat *TaCCR* transcript |
| TACCR-Q-R | 5'-GTGCCCTTGACGGTGTAG-3' |  |
| TaCOMT1-Q-F | 5'-CATCTACGCCAACGCATT-3' | Q-RT-PCR wheat *TaCOMT1* transcript |
| TaCOMT1-Q-R | 5'-GAGGAAACACCAAGCCAAAG-3' |  |
| TaActin-F | 5'-CACTGGAATGGTCAAGGCTG-3' | Q-RT-PCR wheat *Actin* transcript |
| TaActin-R | 5'-CTCCATGTCATCCCAGTTG-3' |  |
| TaCAD12-4166-ZJF | 5’CGCGGAGTACGTGAACAC-3’ | PCR for over-expressing *TaCAD12* wheat |
| Tnos-3214L | 5’-AAAACCCATCTCATAAATAACG-3’ |  |
|  |  |  |
|  |  |  |
