# Supplementary figures and images for: A Wheat Cinnamyl Alcohol Dehydrogenase TaCAD12 Contributes to Host Resistance to the Sharp Eyespot Disease
Source: Front Plant Sci. 2016 Nov 16;7:1723. doi: 10.3389/fpls.2016.01723 (PMC5110560; doi:10.3389/fpls.2016.01723)

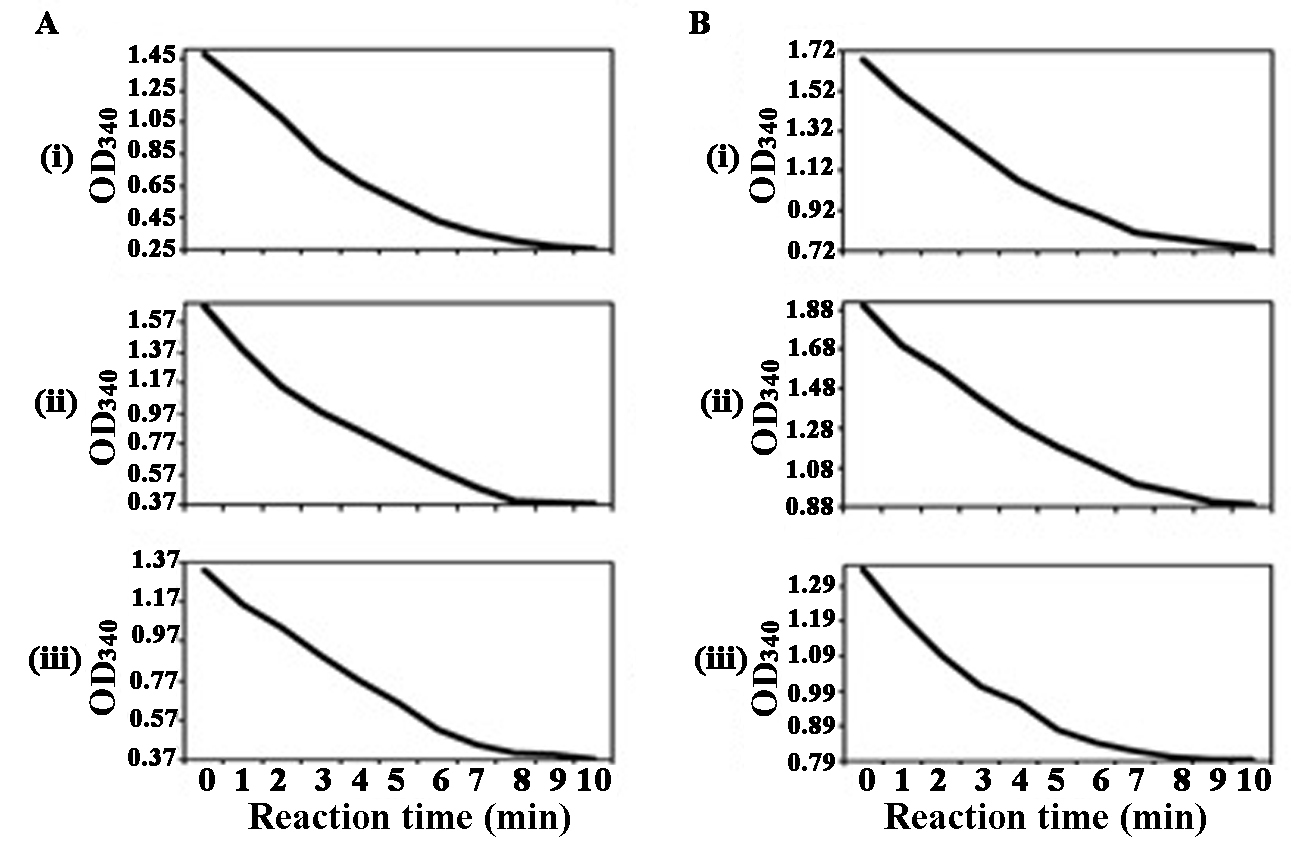

Supplement: Figure S2 — Schematic representation of the barley strip mosaic virus (BSMV) genome organization and BSMV recombinant constructs engineered to express GFP and TaCAD12 fragments as inverted repeats. RNAs α, β, and γ were as described (Holzberg et al., 2002). The 190-bp TaCAD12 fragment (from 1011 to 1200 nt in TaCAD12 cDNA sequence) was sub-cloned in an antisense orientation into the Nhe I restriction site of the RNA γ of BSMV. [file Image_1.JPEG]

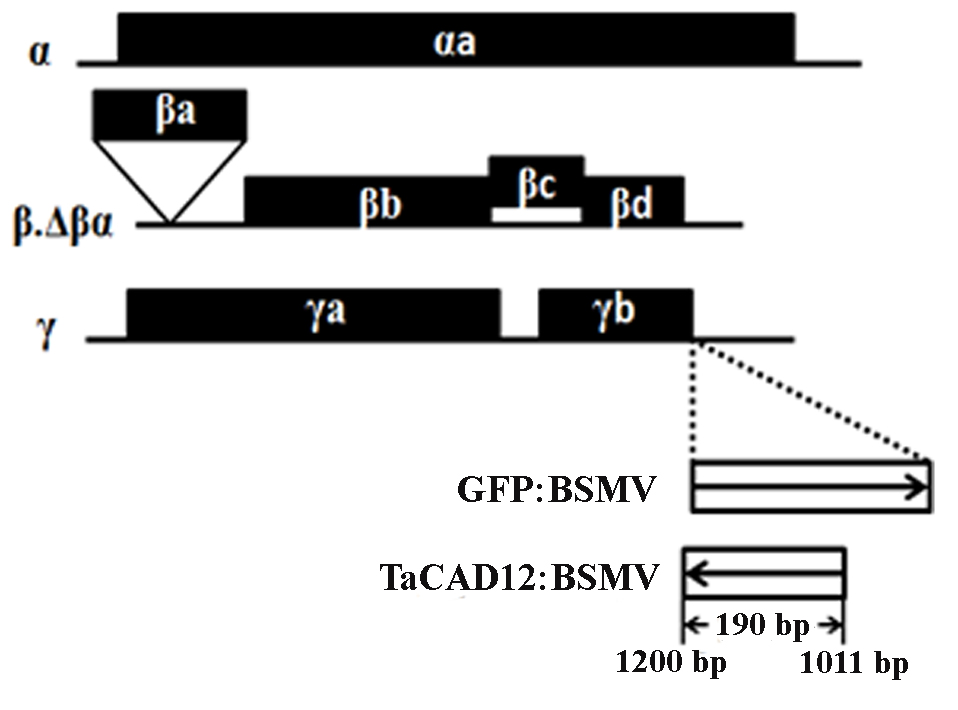

Supplement: Table S1 — Sequences of primers used in this study. [file Image_2.JPEG]
